# Tonsilitis

**Published:** 1888-11

**Authors:** 


TONSILITIS with white exudations on the tonsils is prevalent in this section of Georgia, and now and then a genuine case of diphtheria. We have treated a number of these cases of tonsilitis recently as follows and with good success. First the affected tonsil is touched with solution of nitrate of silver (20 gr. to the ounce) and the following prescriptions alternated at intervals of two hours:

℞ Milk.................................................... ℥ iv,
Spts. Turpentine........................................gtt. xx.

Dose—A teaspoonful every three hours.

℞ Pulv. Borax..........................................
Lac. Sulphur..............................................aa ℥ ss.

A small quantity blown through a quill into the throat.

Give asa purgative at the commencement 5 grs. of sacharated calomel and use during the treatment frictions with turpentine to the external throat. If fever be high give half drop of aconite every two hours.

